# Network Scale-Up Correction Factors for Population Size Estimation of People Who Inject Drugs and Female Sex Workers in Iran

**DOI:** 10.1371/journal.pone.0110917

**Published:** 2014-11-03

**Authors:** Ahmad Maghsoudi, Mohammad Reza Baneshi, Mojtaba Neydavoodi, AliAkbar Haghdoost

**Affiliations:** 1 Regional Knowledge Hub, and WHO Collaborating Centre for HIV Surveillance, Institute for Futures Studies in Health, Kerman University of Medical Sciences, Kerman, Iran; 2 Research Centre for Modeling in Health, Institute for Futures Studies in Health, Biostatistics and Epidemiology Department, Kerman University of Medical Sciences, Kerman, Iran; 3 Faculty of Public Health, Shiraz University of Medical Sciences, Shiraz, Iran; Istituto Superiore Sanita', Italy

## Abstract

**Introduction:**

The results of the network scale-up (NSU) method in estimating the size of key populations for HIV might be biased if the recruited subjects are not fully informed of the risky behaviors of people in their networks (low visibility), or key populations have a smaller social network (low popularity). We aimed to measure such biases in the size estimation of people who inject drugs (PWIDs), and female sex workers (FSWs) in Iran.

**Methods:**

We interviewed 163 male PWIDs, 76 FSWs (known as egos) and 600 subjects from the general population. We selected twenty first-names (ten males and ten females) and asked the study subjects separately how many people they knew with one of these names (known as alters). Visibility Factor (VF) was defined as the percentage of FSW or PWID alters that were aware of their behavior. In addition, the popularity factor (PF) was calculated by dividing the number of alters reported by FSWs and PWIDs into that of the general population. The 95% uncertainty intervals (UI) were calculated using bootstrap technique.

**Results:**

The VF was estimated at 54% (95% UI: 52%–56%) for PWID and 45% (95% UI: 42%– 48%) for FSW. The VF among the peer alters was significantly higher than non-peer ones. The PF for PWID and FSW was 69% (95% UI: 66%–73%) and 77% (95% UI: 72%–83%), respectively. The cross-validation and name splitting analysis showed that our estimates were not influenced by any single name.

**Conclusions:**

Both correction factors, particularly VF were far from one, and NSU results without correction, could lead to up to 4 times underestimation of the sizes. Therefore, applying these coefficients is necessary in NSU projects.

## Introduction

Estimating the population size of hard-to-count subgroups is an important entity in the area of social science as well as in public health. This is especially crucial in the HIV/AIDS context since reliable estimates of the size of key populations are one of the key pieces of information considered by policy makers; as such information could be used for better tracking the epidemic, program planning, advocacy and more effective resource allocations [1]. The need for this information is more prominent in a country like Iran with a concentrated HIV epidemic among people who inject drugs (PWIDs) and with limited resources as a middle income country [2].

It is well known that the risk of transmitting HIV is extremely high among PWIDs and FSWs [3]; however, these key populations are hidden in the community due to the stigma surrounding their behaviors [4]. The stigma and discrimination against such populations is a challenge for direct estimation techniques to approximate their population size. Given their relatively small population size, any survey for direct estimation requires a very large sample size, which is also not often feasible [5]. Such challenges are even more prominent in a setting like Iran, with a dominant Islamic culture, and where stigma around sexual behaviors and its related subpopulations is high.

One promising approach with a practical appeal is the network scale-up method (NSU) [6]. In the NSU, we ask a random sample of the general population how many people they know from the population of interest such as PWIDs in their active social networks [7]. Here, the assumption is that the frequency of the hidden groups in the community is the same as the frequency in the network of the respondents.

Another assumption in the NSU is that the respondents are aware of the sensitive behaviors of people in their active networks [7], [8], [9], [10]. However, justification of this assumption is difficult because of transmission error, a concept implying that sensitive information is not always transmitted. This error results in an underestimation of the size of those target populations [6], [11].

In addition, we assume the network size of the members of the hidden and general populations to be the same. However, it may not always be the case and the network size of the populations may differ, introducing a concept called the relative network size [12]. For instance, the network size of the HIV patients might be one third of that of the general population [13]. This indicates that the key populations might have smaller networks, possibly due to the stigmatized nature of their behaviors.

Therefore, the transmission error and relative network size should be taken into account for adjusting NSU crude estimates. So far, only a few studies conducted in Ukraine, Brazil, Rwanda, and Japan have measured and reported such correction factors [5], [14], [15], [16], and none of these could be generalized to high-stigmatized settings like Iran or other neighboring countries in the Eastern Mediterranean region. Therefore, we conducted this study to measure the visibility and popularity factors for two main key populations, PWIDs and FSWs, who have been disproportionately affected by a HIV epidemic in Iran.

## Methods

The study was carried out in two large cities of Iran, Shiraz and Kerman, both located in the south of the country. Using facility based sampling, PWIDs and FSWs, who had already received services from three centers, were recruited (163 male PWIDs, 79 in Kerman and 84 in Shiraz; and 76 FSWs in Shiraz). These centers were the referral sites where visitors from all over the cities converged. Our trained interviewers visited the centers at different working hours from early in the morning to evening, during the whole week. They explained the objectives of the study to the eligible subjects and addressed their questions before the formal interview.

The eligible PWIDs (men who had injected drugs at least once in the past year) and FSWs (women who had sold sex at least once in the past year) were enrolled in the survey. Due to cultural issues, we were concerned that requesting a written consent form would lead to a low response rate or incorrect answers [17]. Therefore, a verbal consent was taken from the participants. The study protocol and procedures of the participants' consents were reviewed and approved by both the Research Review Board, and the Research Ethical Committee at Kerman University of Medical Sciences.

Using a structured face-to-face interview, data were collected by a trained same-sex interviewer. Moreover, in a street-based study, a random sample of 600 participants (300 in Shiraz and 300 in Kerman) were recruited from the general population. Eligible people over eighteen years of age were selected from three public areas in each city; the locations were chosen from low, middle and high socio-economic districts in Kerman and Shiraz. To achieve a representative sample, we interviewed pedestrians of different age and sex groups. Those subjects who provided verbal informed consent were then interviewed as explained above.

### Selection of names for estimation of VF and PF

We selected ten one-part male and ten one-part female first-names with a frequency of 0.1% to 4% in the community to maximize the precision of our estimation [5]. This range of prevalence was selected, as recommended in the literature, because it has been shown that respondents usually overestimate small populations, and underestimate large ones. This error is known as recall bias. We did not use two-part names, or the names that were known to have periodic fluctuations and also names used for both males and females. We also applied a standard definition for ‘know’ as ‘People whom you know and who know you, by name, with whom you can interact, if needed and with whom you have contacted over the last two years personally or by telephone, or via email [18]. The questionnaires were not specifically randomized by names; instead, we put the male names in the first place followed by the female names.

### Estimation of Correction Factors

In this study, we used the methodology outlined in a Brazil paper and a Ukraine report [1], [5], [6]. In addition, we calculated the correction factors classified by the risk behaviors of alters. Furthermore, we implemented random effects mixed model analysis to explore to what extent the variations seen in the replies were associated with the names selected. Finally leave one out cross validation and name splitting approaches were applied to address the internal validity of our results.

### Visibility Factor (VF)

**Figure 1:**
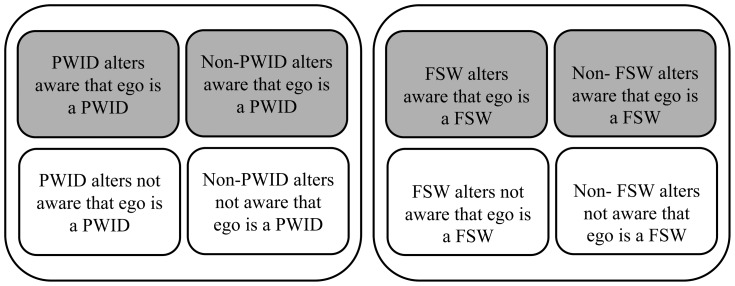
The four strata for collecting the number of alters in PWID and FSW survey.

In brief, VF is the percentage of FSW or PWID alters that were aware of their behaviors, i.e. selling sex or injecting drugs. To measure VF, we asked every recruited subject (known as egos) the total number of their alters with any of the twenty selected names (reference group), and of those, how many were aware that the ego was a member of FSW or PWID subpopulations. These numbers were also stratified as if the alters themselves were or were not FSW/PWID (Figure 1).

The above table was calculated for each respondent and for each name. For each name, replies of all subjects were aggregated. Using formula 1, the VF was calculated by dividing the total number of aware alters 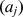 (grey boxes in Figure 1) by the total number of alters 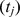 (where j stands for name (from 1 to 20).

Formula (1): 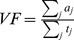


Additionally, we calculated VF for each of the respondents and for each name separately 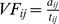, where i and j represent respondent and name. For example, for 163 PWIDs, we calculated 3260 VFs (163 multiplied by 20). These 3260 VFs were considered to be the outcome. We then developed a random effects mixed model analysis. We treated names as a random factor to explore to what extent a variation seen in VFs was associated with the names.

### Popularity Factor (PF)

Briefly, the PF is the ratio of the average networks size of FSWs or PWIDs to the network size of the general population. For each of the twenty selected names, we calculated the PF separately. To do so, for each name, the average of alters known by target group 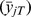 was divided by that of the general population 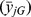. This indicates that 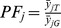. Finally, the average of these 20 PFs was considered as the final PF. Formula 2 illustrates this concept as an equation [16]:

Formula (2): 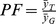


In order to estimate the proportion of variance in the number of alters known by each respondent which contributed to the estimation of PF, we modeled 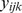
where 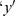 represented the number of known alters, 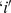 the respondents, 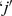 the selected names, and 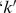 the group (high risk group versus general population). In a random effect mixed model, we treated the selected names as a clustering factor. This approach was different from the one applied to the model VF. It was possible to extract the numerator and the denominator of VF for each respondent and for each name separately. However, this was not possible for the PF, since the numerator and the denominator were computed based on the responses of high risk groups and general populations, respectively.

### Construction of Uncertainty Interval (UI)

To estimate the 95% uncertainty intervals (UI) for VF and PF, we applied bootstrap procedure. We drew 1000 independent samples, with replacement, from the original data. In each of the bootstrap sample, 20 statistics (one for each name) and its average was calculated as the best estimate of that bootstrap sample. The derived statistics were sorted and the percentiles 2.5% and 97.5% were considered as the lower and upper bounds of UI.

### Validation process

To assess the impact of each name in our estimates, we followed two approaches: leave-one-out cross validation and name splitting. In the leave one out cross validation, each name was deleted in turn and statistics were estimated using the remainder of the nineteen names. Therefore, twenty statistics were computed. The average of these values was computed and compared with the full results (based on twenty names).

In the name splitting approach, we randomly divided twenty names in to two equal sets. Each set contained five male and five female names. Statistics were computed and compared in each set independently.

## Results

The mean age of the participants selected from the general population was 30.5 years; half were male, and 7% were unemployed. All PWIDs were male with a mean age of 37.4 years. Around 10% of them had an academic degree and 27.6% were unemployed. The mean age of the FSWs was 32.1 years; 17% were single, 33% were married, and 50% were widowed or divorced.

### Visibility Factor

#### PWID group

In total, 163 PWIDs had 2596 alters with one of the selected first names, giving an average number of alters with one of the selected names of 15.92 persons. The total number of alters aware of the risky behaviors of egos was 1409; the estimated VF was 54% (95% UI: 52%, 56%), (Table 1). This implies that, on average, 54% of the people in the network of a PWID were aware that the ego was a PWID.

**Table 1:** Estimation and validation of visibility factor for PWID and FSW groups.

		Estimation phase			Validation phase			Sensitivity phase	
		All alters	Peer alters	Non-peer alters	Cross Validated statistics	First set of names	Second set of names		
					All alters	All alters	All alters	Male alters	Female alters
Statistics	Group	10 M, 10 F	10 M, 10 F	10 M, 10 F	10 M 10 F (19 used at each step)	5 M, 5F	5 M, 5F	10 M	10 F
VF	PWID	54% (52%, 56%)	88% (79%, 99%)	40% (36%, 44%)	55%	53%	55%	58% (56%, 61%)	46% (42%, 50%)
	FSW	45% (42%, 48%)	76% (64%, 89%)	34% (30%, 39%)	45%	43%	45%	42% (38%, 46%)	48% (44%, 53%)

M: Male names, F: Female names.

VF: Visibility Factor.

Furthermore, on average, 30% of alters of a PWID were a PWID, themselves. The VF among the PWIDs and non-PWIDs' alters were significantly different (88% (95% UI: 79%, 99%) vs. 40% (95% UI: 36%, 44%)). In other words, compared with non-PWID's alters, the PWID's alters were more likely to be aware of the behaviors of the egos (Table 1).

**Figure 2:**
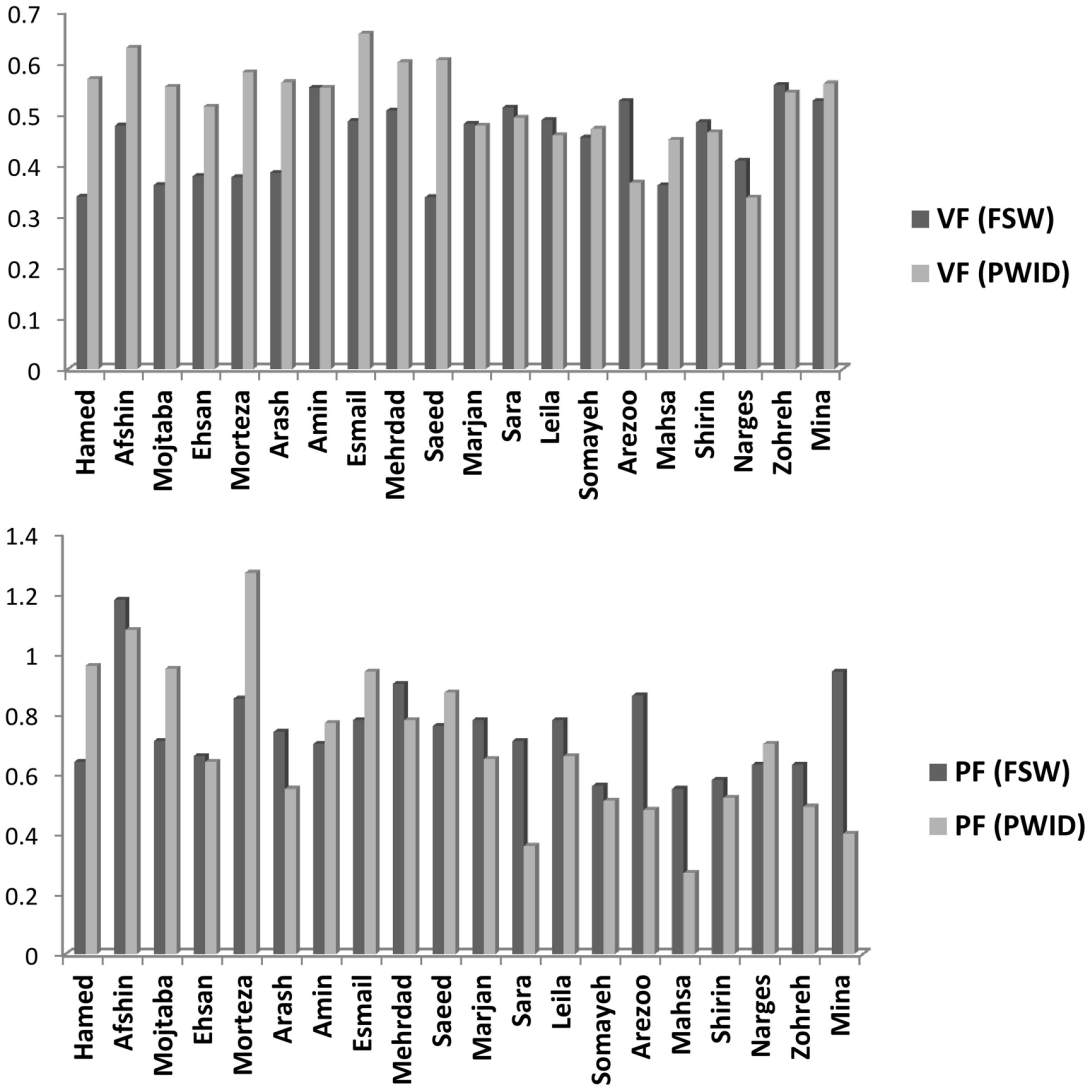
Estimation of Visibility Factor (VF), (top panel), and Popularity Factor (PF), (bottom panel), based on all 20 names.

Calculating the VF based on different names separately (Figure 2), we have found that VF ranged from 34% to 66% (52% to 66% based on 10 male names, and 34% to 56% based on 10 female names). We have also observed that the VF based on ten male and ten female alters was 58% (95% UI: 56%, 61%) and 46% (95% UI: 42%, 50%) respectively, which significantly differed (Table 1). This indicates that the visibility of this behavior amongst the male alters was higher than that of the female alters.

Leave one out cross validated VF for PWID group was 55%. In the name splitting approach, the VF in the first and second sets of the names were estimated at 53% and 55% (Table 1).

The results of the mixed model revealed that 50% of variation in the VFs (for each respondent for each name) was explained by the selected names.

#### FSW group

In the FSW group, 76 subjects had 1316 alters with one of the selected first-names; which means that on average, each FSW had 17.31 alters.

The total number of the aware alters was 590. The VF in FSW was 45% (95% UI: 42%, 48%). Furthermore, about one fourth of alters of FSWs were a FSW themselves. The VF among FSWs alters was significantly higher than the non-FSWs alters (76% (95% UI: 64%, 89%) vs. 34% (95% UI: 30%, 39%)), (Table 1).

Having calculated the VF for each name (Figure 2), we found that VF ranged from 34% to 56% (34% to 55% in 10 male names, and 36% to 56% in 10 female names). The VF based on 10 male and 10 female names was 42% (95% UI: 38%, 46%) and 48% (95% UI: 44%, 53%) respectively (Table 1). The difference did not reach a level of statistical significance, indicating that the visibility of this behavior among male and female alters was similar.

Leave one out cross validated VF for FSW group was 45%. Furthermore, the VFs in the first and second sets of names were 43% and 45% (Table 1). The results of the mixed model revealed that 60% of variation in the VFs (for each respondent for each name) was associated with the names selected.

### Popularity Factor

#### PWID group

We estimated the PF of the PWIDs at 69% (95% UI: 66%, 73%), (Table 2). This indicates that the network of a PWID was significantly smaller than that of the general population. Having analyzed ten male and ten female names separately, the PF was estimated at 88% (95% UI: 83%, 94%) and 50% (47%, 55%), which was of statistical significance (Table 2).

**Table 2:** Estimation and validation of popularity factor for PWID and FSW groups.

Statistics	Group	Estimation phase			Validation phase			Sensitivity phase	
		All alters	Peer alters	Non-peer alters	Cross Validated statistics	First set of names	Second set of names	Male alters	Female alters
		10 M, 10 F	10 M, 10 F	10 M, 10 F	All alters	All alters	All alters		
					10 M 10 F (19 used at each step)	5 M, 5F	5 M, 5F	10 M	10 F
PF	PWID	69% (66%, 73%)	NA	NA	70%	63%	75%	88% (83%, 94%)	50% (47%, 55%)
	FSW	77% (72%, 83%)	NA	NA	74%	72%	77%	79% (73%, 89%)	70% (67%, 82%)

M: Male names, F: Female names.

PF: Popularity Factor.

NA: Not Applicable.

The results of leave one out cross validation showed that the PF for PWID group was 70%. This statistics in the first and second sets of names were 63% and 75% (Table 2).

In addition, having calculated the PF for each name (Figure 2), we witnessed that the PF ranged from 27% to 127% (55% to 127% based on 10 male names, and 27% to 70% based on 10 female names). The results of the mixed model revealed that 67% of variation in the number of alters known by respondents for each name was associated with the names selected.

#### FSW group

The PF of FSWs was also 77% (95% UI: 72%, 83%), (Table 2). This indicates that the PF of FSWs was significantly lower than that of the general population. Using 10 male names, the PF was estimated at 79% (95% UI: 73%, 89%). The corresponding figure of 10 female names was 70% (95% UI: 67%, 82%), (Table 2).

The results of leave one out cross validation showed that the PF for FSW group was 74%. Once the values were estimated for each of the two name sets, the PF reached 72% and 77% (Table 2).

Moreover, having calculated the PF for each name (Figure 2), we saw that the PF ranged from 55% to 118% (64% to 118% based on 10 male names, and 55% to 94% based on 10 female names). Furthermore, the results of the mixed model revealed that 69% of variation in the number of alters known by the respondents for each name was associated with the names selected.

## Discussion

In this study, we calculated the visibility and popularity factors of the Iranian FSWs and PWIDs. It should be noted that we aimed to calculate two correction factors needed to adjust the NSU results.

We found that the VF for the PWIDs and FSWs was considerably lower than one. Only approximately half of the alters were aware of the behaviors of PWID's egos. As for the FSWs, such a percentage was even lower (45%). The visibility among the male and female alters of PWISs differed, implying that within their networks, the males were more aware of their behaviors (58% versus 46%). However, such a large and significant difference was not observed between male and female alters in the network of FSWs (42% versus 48%). In both groups, as we may expect, the visibilities among peer alters were considerably higher (PWIDs: 88% versus 40%; FSWs: 76% versus 34%), which means that in both groups, the subjects might reveal their risky behaviors to those alters who were practicing themselves.

In addition, social network size of these two groups was approximately two third of the general population. In the PWID group, based on male names, the difference between PF and 1 was of marginal significance, indicating that the male portion of the network size of the PWID group was more or less the same as that of the general population. However, the female portion of the network size of PWIDs was about half of the general population. On the other hand, both male and female portions of the network of FSWs were smaller than that of the general population.

Furthermore, our results of the random effects revealed that most of the variations in the subjects' replies were associated with the selected names. On the other hand, leave one out cross validation and name splitting analysis suggested that our estimates were not influenced by a single name.

All in all, this evidence suggests that it is necessary to select both names from both genders, and to select a sufficient number of names to obtain robust statistics. Therefore, at least twenty names are needed when using this methodology

Given the fact that selling of sex or injecting drugs are stigmatized and punishable acts in many communities/countries, including Iran, the visibility of those involved in these acts is expected to be low [19], [20], [21]. The invisibility of such behaviors could severely affect the accuracy of the NSU estimation regarding the population size of the key populations of HIV. In addition, because of the differences in these coefficients for male and female alters, we strongly recommend that the size of the key populations is computed in each gender separately in NSU studies so that the results can be adjusted using gender specific correction factors.

In a study on heavy drug users in Brazil, the social visibility was estimated at 77% [6], which is much higher than the one observed in our study (54%). Such a difference might be due to the different levels of stigma experienced by the members of these two high risk groups, or because they explored “heavy drug users”, which might be more visible to the community than other types of drug users.

In 2009 in Ukraine, 28 PWIDs, 21 FSW, and 108 Men who have Sex with Men (MSM) were interviewed using the same methodology that we applied. The social visibility of PWIDs, FSWs, and MSM was 57%, 34%, and 24%, respectively. The reported visibility factor of PWIDs is comparable to our findings, while the visibility for FSW was lower than ours. It could be partly explained by socio-cultural differences, but due to the small sample size of the Ukraine study, it may not be absolutely ideal for comparison.

Similar to our findings, other studies have also reported higher visibility for PWIDs [5], [6]. Drug injection is likely to change the appearance of users which could make them more visible in societies.

Furthermore, our results showed that amongst PWIDs and FSWs, the within group VF was around twice higher. In other words, the risky behavior of this group was less visible to the general population, compared to alters who were members of the target population. This finding is not unique, as usually individuals of a subpopulation, know more about each other and have more information about their behaviors.

The PF of the PWIDs in Iran and heavy drug users in Brazil were quite similar (70% vs. 69%) [16]. As expected, the popularity of FSWs was slightly higher than PWIDs (77% vs. 69%). FSWs might have a larger social network due to the nature of their job. On the other hand, PWIDs might have less connection with the community perhaps due to personal preferences or due to restrictions from the community making them more isolated and marginalized. This makes PWIDs even less accessible in the community and through the networks.

Since the application of NSU technique in the estimation of the size of sub-groups has been a hot topic over recent years, more attention to its biases and in exploring practical solutions to verify the crude results of this technique is necessary. Our study is one of the few published papers in this area, which not only estimated the two main correction factors for PWIDs and FSWs, but also presented new concepts in the analysis and validation of findings; this could be applied in further studies in other settings.

In estimation of PF, Salganik et al. calculated the ratio of averages. However, we estimated the PF for each name independently and considered its mean as the final estimate. We thought that each name can be considered as a subsample to estimate PF. This is the natural way to consider sampling variation in the analysis. In our approach, the contribution of all names in mean calculation is equal to 1. In Salganik's approach, those names with a higher frequency would get a higher weight. Although we are not in the position to discuss which one provides a more robust estimate, we yet believe that there is no rational to give a higher weight to names with a higher frequency. We believe that more theoretical work is required to compare performance of these two different approaches. On the other hand, to address the impact of weighting scheme on our estimates, we applied Salganik's approach. The results of ratio of averages were 70% and 76% for IDU and FSW respectively, which were almost the same as the average of ratios (69% and 77%). Therefore, we trust that our estimates are not affected by weighting scheme.

Hereby, we acknowledge the main limitation of our study that is the selection bias that could occur through the non-probability recruitment of the study subjects from target groups. We tried to address a part of this by recruiting from different (both public and private) centers which provided services to diverse subpopulations of PWIDs and FSWs. Although our findings may not be easily generalized to all PWIDs and FSWs in Iran, we consider that by recruiting at least a portion of the target groups (the most visible section), our value for the VFs would somehow overestimate the visibility correction factor and underestimate the relative network size (as marginalized, unlinked to services, key populations might have smaller networks). These need to be explored in further confirmatory studies to measure the magnitude of this bias in our estimates.

One more issue to be addressed in future studies is the name order effect. We randomly sorted the list of male and female names, but all male names were listed before female names. Although based on the findings, we could infer that the selection of names and their numbers are significant, particularly in the estimation of the PF, more investigation is needed to explore the order effect of names in the results.

## Conclusions

Based on our findings, the results of NSU size estimations need to be adjusted for both visibility and popularity biases; otherwise the findings may underestimate the size of the hidden groups, substantially (3 to 4 times). Such biases are even more prominent in the size estimation of FSWs. Based on our findings; we can conclude that the correction of NSU studies for the level of visibility is relatively more important than relative network size.

## References

[1] Salganik MJ, Mello MB, Abdo AH, Bertoni N, Fazito D, et al (2011) The game of contacts: estimating the social visibility of groups. Social networks 33: 70–78. 21318126 10.1016/j.socnet.2010.10.006 PMC3035387

[2] Shokoohi M, Baneshi MR, Haghdoost AA (2011) Estimation of the active network size of Kermanian Males. Addiction and Health 2: 81–88. PMC3905511 24494105

[3] Bernard HR, Hallett T, Iovita A, Johnsen EC, Lyerla R, et al (2010) Counting hard-to-count populations: the network scale-up method for public health. Sexually transmitted infections 86: 11–15. 10.1136/sti.2010.044446 PMC3010902 21106509

[4] Shokoohi M, Baneshi MR, Haghdoost A-a (2012) Size estimation of groups at high risk of HIV/AIDS using network scale up in Kerman, Iran. International journal of preventive medicine 3: 471–477.. 22891148 PMC3415187

[5] Paniotto V, Petrenko T, Kupriyanov O, Pakhok O (2009) Estimating the size of populations with high risk for HIV using the network scale-up method. Ukraine: Kiev International Institute of Sociology.

[6] Salganik MJ, Fazito D, Bertoni N, Abdo AH, Mello MB, et al (2011) Assessing network scale-up estimates for groups most at risk of HIV/AIDS: evidence from a multiple-method study of heavy drug users in Curitiba, Brazil. American journal of epidemiology 174: 1190–1196. 22003188 10.1093/aje/kwr246 PMC3208143

[7] Stroup DF (2010) Size Estimations for High Risk Groups Moldova. Chisinau: Unpublished.

[8] McCarty C, Killworth PD, Bernard HR, Johnsen EC, Shelley GA (2001) Comparing two methods for estimating network size. Human Organization 60: 28–39.

[9] Russell Bernard H, Johnsen EC, Killworth PD, Robinson S (1991) Estimating the size of an average personal network and of an event subpopulation: Some empirical results. Social science research 20: 109–121.

[10] Jackson D, Kirkland J, Jackson B, Bimler D (2005) Social Network Analysis and Estimating the Size of Hard-to-Count Subpopulations1. Connections 26: 49–60.

[11] Killworth PD, Johnsen EC, McCarty C, Bernard HR, Shelley GA (2003) Attempting to quantify transmission and barrier errors in scale-up methods. preparation; Citeseerx Website. Available: http://nersp.nerdc.ufl.edu/~ufruss/documents/trans.pdf. Accessed 2014 Sep 30.

[12] UNAIDS (2012) Consultation on Network scale-up and other size estimation methods from general population surveys. Epidem Website. Available: http://www.epidem.org/sites/default/files/reports/Consultation%20on%20network%20scale-up.pdf. Accessed 2014 Sep 30.

[13] Shelley GA, Bernard HR, Killworth P, Johnsen E, McCarty C (1995) Who knows your HIV status? What HIV+ patients and their network members know about each other. Social networks 17: 189–217.

[14] Dhsprogram Website. Estimating the size of populations through a household survey (ESPHS) Rwanda 2011, 2012. National University of Rwands SoPHIoHIVADP, Control JUNPoHIVAICFIMDHS. Available: http://dhsprogram.com/pubs/pdf/FR261/FR261.pdf. Accessed 2014 Sep 30.

[15] Ezoe S, Morooka T, Noda T, Sabin ML, Koike S (2012) Population size estimation of men who have sex with men through the network scale-up method in Japan. PloS one 7: e31184. 22563366 10.1371/journal.pone.0031184 PMC3341925

[16] Salganik MJ, Fazito D, Bertoni N, Abdo AH, Mello MB, et al (2011) Web Appendix Assessing network scale-up estimates for groups most at risk for HIV/AIDS: Evidence from a multiple method study of heavy drug users in Curitiba, Brazil. American journal of epidemiology 174: 1190–1196. 22003188 10.1093/aje/kwr246 PMC3208143

[17] Zolala F (2011) Exploring routine data collection systems in Iran, focussing on maternal mortality and using the city of Bam as a case study: The University of Edinburgh.

[18] McCormick TH, Salganik MJ, Zheng T (2010) How many people do you know? Efficiently estimating personal network size. Journal of the American Statistical Association 105: 59–70. 23729943 10.1198/jasa.2009.ap08518 PMC3666355

[19] Abadía-Barrero CE, Castro A (2006) Experiences of stigma and access to HAART in children and adolescents living with HIV/AIDS in Brazil. Social science & medicine 62: 1219–1228. 16099573 10.1016/j.socscimed.2005.07.006

[20] Parker R, Aggleton P (2003) HIV and AIDS-related stigma and discrimination: a conceptual framework and implications for action. Social science & medicine 57: 13–24. 12753813 10.1016/s0277-9536(02)00304-0

[21] Johnston LG, Sabin K (2010) Sampling hard-to-reach populations with respondent driven sampling. Methodological Innovations Online 5: 38–48.

